# OTSC combined with jejunal mucosal ESD for closure of jejunal dilated blind pouch and anastomotic metal stent placement in the treatment of post-oesophagojejunostomy stricture: a case report (with video)

**DOI:** 10.3389/fmed.2026.1881491

**Published:** 2026-08-28

**Authors:** Dan Wu, Da Yong Sun, Xiang Yu Wang, Hong Wei

**Affiliations:** Department of Gastroenterology, The Second People's Hospital of Shenzhen City (The First Affiliated Hospital of Shenzhen University), Shenzhen, China

**Keywords:** case report, covered metal stent, ESD, jejunal dilated blind pouch, oesophagojejunostomy stricture, OTSC

## Abstract

**Rationale:**

Anastomotic stricture after oesophagojejunostomy occurs in 5%−15% of cases, and those complicated by jejunal dilated blind pouch are clinically rare. Traditional single treatments cannot address both lesions simultaneously. The combination of over-the-scope clip (OTSC), endoscopic submucosal dissection (ESD), and covered metal stent provides a potential minimally invasive approach for such complex postoperative complications.

**Patient concerns:**

A 66-year-old male with a 5-year history of total gastrectomy for cardiac cancer developed progressive dysphagia 3 months after surgery for lower esophageal squamous cell carcinoma. He had difficulty with semiliquid food, accompanied by acid reflux and nocturnal cough, with an approximately 10-kg weight loss that severely impacted his eating function and quality of life. He worried about symptom progression and reoperation trauma.

**Diagnoses:**

Definitive diagnoses included benign oesophagojejunostomy anastomotic stricture, jejunal dilated blind pouch distal to the anastomosis, and histories of lower esophageal squamous cell carcinoma surgery and cardiac cancer total gastrectomy. Pathology excluded tumor recurrence. Computed tomography (CT) and endoscopy confirmed a 2.4cm-long strictured segment (0.7 cm diameter) as measured on axial CT imaging at the level of the aortic arch using electronic calipers and a 3.0 cm × 2.5 cm jejunal blind pouch (maximum dimensions on axial CT slice).

**Interventions:**

A two-step endoscopic procedure was performed: 1) Jejunal blind pouch closure: ESD resected mucosal tissue to reduce volume, followed by OTSC placement to close the orifice; 2) Anastomotic covered metal stent placement: After balloon dilation of the stricture to 1.5 cm, a 2.0 cm × 8.0 cm covered metal stent was implanted. Perioperative management included anti-infection, nutritional support, and acid suppression therapy.

**Outcomes:**

The patient tolerated liquid food on postoperative day 1 and semiliquid food on day 3. One month later, he ate soft food normally with a 3-kg weight gain, and reexamination showed well-positioned stent and closed blind pouch. At 3-month follow-up, the stent was removed without restenosis or blind pouch recurrence. At 9-month follow-up, the patient remained asymptomatic with no recurrent dysphagia, and his self-reported weight had further increased to 70.3 kg (total gain 10.3 kg from baseline). No serious complications occurred within 9 months, and his quality of life improved significantly.

**Lessons:**

For “stricture + blind pouch” cases, treating the blind pouch first avoids reflux affecting stent efficacy. ESD pretreatment enhances OTSC closure safety, covered stents dilate strictures, and refined perioperative management reduces complications. This case represents preliminary experience suggesting the regimen's potential feasibility in carefully selected patients. However, the evidence level remains limited, and long-term efficacy and safety require validation through prospective, multi-center studies with larger sample sizes.

## 1 Introduction

Esophageal cancer is a common malignant tumor of the digestive tract in China. Surgical resection combined with digestive tract reconstruction is the main treatment modality. For patients with a previous history of subtotal gastrectomy, oesophagogastric reconstruction cannot be performed; thus, oesophagojejunostomy has a wide range of indications (1, 2). However, anastomotic stricture is a common postoperative complication, with an incidence ranging from 5 to 20%, and severely affects patients' eating function and quality of life (3, 4). Some patients may also develop rare complications, such as jejunal dilated blind pouch distal to the anastomosis, because of intestinal content retention and intestinal motility disorders caused by stricture, further increasing the difficulty of treatment (5, 6). The incidence of anastomotic stricture after esophagojejunostomy ranges from 5 to 15%, with higher rates in patients undergoing multiple foregut procedures (3, 4, 7).

In traditional treatment, endoscopic balloon dilation and bougie dilation are typically used for anastomotic stricture. However, for patients with severe stricture, the long-term recurrence rate can exceed 40% (8, 9). Although stent placement can improve the patency maintenance rate, simple stent placement cannot solve problems such as food retention and reflux caused by a jejunal dilated blind pouch (10). Although traditional surgical treatment can simultaneously address both lesions, it is associated with major surgical trauma and slow recovery. In postoperative patients, the surgical risk is significantly increased (11). Reoperation in patients with prior upper gastrointestinal surgery carries significant morbidity. Studies report anastomotic leak rates of 8–15%, wound infection rates of 10–20%, and prolonged ileus in 15–25% of cases following redo foregut surgery (12, 13).

As an endoscopic closure device, OTSCs have definite efficacy in the repair of gastrointestinal perforations and fistulas because of their strong ability to grasp and close tissue (14). ESD can accurately resect mucosal tissue to create favorable conditions for lesion closure (15). On this basis, in this case, OTSC combined with jejunal mucosal ESD was adopted to close the jejunal dilated blind pouch, and a covered metal stent was simultaneously implanted at the anastomosis site to treat a patient with severe stricture and jejunal blind pouch dilation after oesophagojejunostomy, achieving good results. This report aims to provide a reference for the clinical treatment of similar cases.

## 2 Clinical data

### 2.1 General information

A 66-year-old male patient was admitted to the hospital because of “progressive dysphagia for 3 months.” Five years prior, he had undergone radical total gastrectomy for cardiac cancer at another hospital, and pathology indicated moderately to well-differentiated adenocarcinoma. He received regular chemotherapy after surgery, but the specific treatment details were unknown. Six months prior, he underwent laparoscopic radical oesophagectomy (Ivor–Lewis procedure) + oesophagojejunostomy for “esophageal cancer” at another hospital. Prior to referral to our center, the patient had undergone endoscopic balloon dilation twice at the original surgical hospital (at 4 and 5 months postoperatively, respectively). Each dilation used a controlled radial expansion balloon (CRE, Boston Scientific) to 12 mm diameter for 3 min per session. However, dysphagia recurred within 2–3 weeks after each procedure, with the stricture diameter reverting to < 0.8 cm on follow-up endoscopy. No bougie dilation was attempted. These failed conventional treatments prompted referral for combined endoscopic intervention.

Postoperative pathology revealed moderately differentiated squamous cell carcinoma of the lower esophagus invading the submucosa, with no vascular or neural invasion, no regional lymph node metastasis (0/12), and negative surgical margins. He was followed up regularly after surgery without radiotherapy or chemotherapy. Three months after surgery, he developed dysphagia without obvious inducement, initially manifesting as obstruction when eating solid foods such as steamed buns and rice. The symptoms were relieved after he switched to a soft diet but then progressively worsened, with obstruction even when he consumed liquid food, accompanied by acid reflux and nocturnal reflux cough. He was unable to eat normally, and his weight decreased by approximately 10 kg compared with his peak weight after surgery. This weight loss occurred over a 2-month period immediately preceding admission. He was admitted to our hospital for further treatment. Physical examination at admission revealed a temperature of 36.5 °C, a pulse of 90 beats/min, a respiratory rate of 18 breaths/min, and a blood pressure of 112/60 mmHg. He was conscious and in fair spirits, with moderate nutrition, an emaciated body type, and mild pale complexion. No yellowing of the skin or mucous membranes was observed, and no enlarged superficial lymph nodes were palpable. Cardiopulmonary auscultation revealed no abnormalities, with a regular heart rate of 90 beats/min. The abdomen was flat and soft, without abdominal wall varices, tenderness, rebound tenderness, or muscle tension. The liver and spleen were not palpable below the costal margins, shifting dullness was negative, bowel sounds were 4 times/min with normal pitch, and no oedema of the lower extremities was noted.

### 2.2 Auxiliary examinations

Routine laboratory examinations revealed a white blood cell count of 3.861 × 0^9^/L, a neutrophil percentage of 62.3%, a hemoglobin concentration of 108 g/L, and a platelet count of 2,341 × 0^9^/L. Biochemical indicators included an albumin concentration of 36.5 g/L and a prealbumin level of 0.126 g/L; liver and kidney function, electrolyte, and amylase levels were within the normal range; tumor marker (CEA, CA199, SCC, and CYFRA211) levels were within the normal reference range; and coagulation function and infectious disease screening (hepatitis B, hepatitis C, syphilis, and HIV) results were normal.

Enhanced chest + abdominal CT imaging revealed significant stenosis of the oesophagojejunostomy anastomotic orifice, with a strictured segment length of approximately 2.4 cm (measured on the axial CT image at the level of the aortic arch using electronic calipers, with a window width of 400 HU and window level of 40 HU) and a lumen diameter of approximately 0.7 cm. Proximal esophageal dilation with a small amount of effusion and thickening of the esophageal wall was observed; a dilated blind pouch of the jejunum was seen on the ipsilateral side of the proximal anastomosis, measuring approximately 3.0 cm × 2.5 cm, with a thin wall and a small amount of fluid density inside. Mild adhesion between the blind pouch and the adjacent peritoneum was noted, with no ascites or distant metastasis. The jejunal blind pouch dimensions were measured on the axial slice showing maximum transverse and anteroposterior diameters.

**Figure 1:**
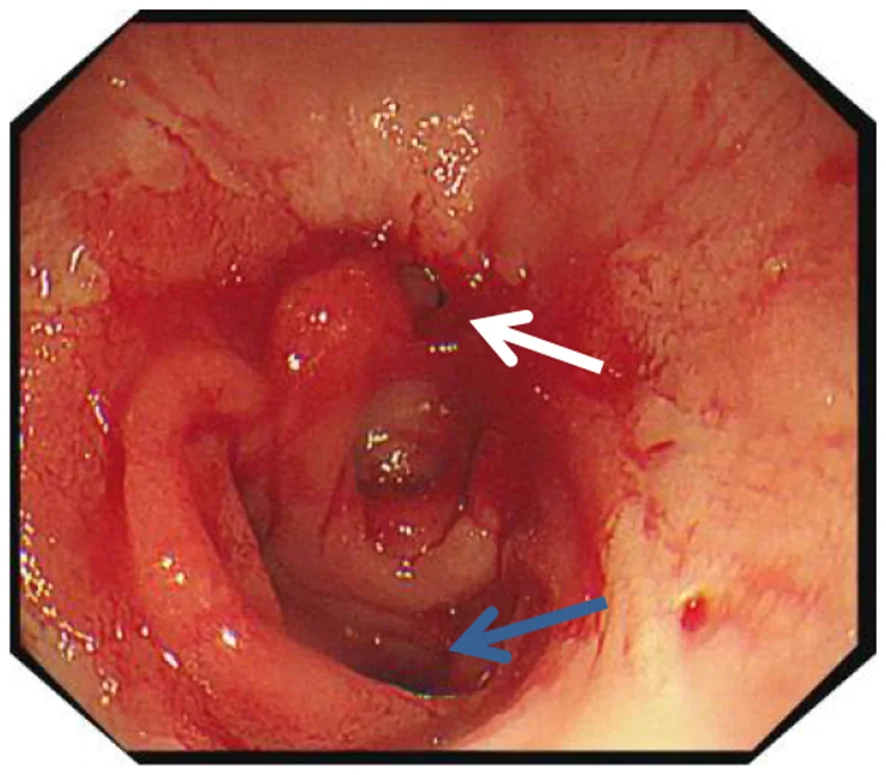
Endoscopic findings showing anastomotic stricture located approximately 30 cm from the incisors. The stricture had a diameter of about 0.7 cm, and the endoscope could barely pass through. The white arrow indicates the anastomotic stricture, and the blue arrow indicates the jejunal blind pouch.

Endoscopic examination: an electronic gastroscope (Olympus GIF-H290Z) was inserted through the oral cavity, and anastomotic stricture was observed approximately 30 cm from the incisors, with a diameter of approximately 0.7 cm, and the endoscope barely passed through (Figure 1); the esophageal mucosa proximal to the stricture was congested and oedematous, with a small amount of food residue and mucus retention on the surface and scattered erosions on the mucosa; three pieces of mucosal tissue at the stricture were taken with endoscopic forceps for pathological examination. The pathological results indicated chronic mucosal inflammation with squamous epithelial hyperplasia, focal lymphocytic infiltration, and submucosal fibrosis; no atypical cells, dysplasia, or tumor cells were found. Immunohistochemistry (CK7, CK20, CDX2) confirmed squamous epithelial origin without intestinal metaplasia, definitively excluding tumor recurrence. A small amount of food residue was seen in the dilated jejunal blind pouch on the ipsilateral side of the proximal anastomosis. After flushing, the mucosa was intact and congested, with no ulcers, bleeding, or neoplasms, and the peristalsis of the intestinal tube around the blind pouch was normal.

### 2.3 Diagnosis and condition analysis

On the basis of the patient's surgical history, clinical manifestations, and auxiliary examinations, the definitive diagnoses were as follows: 1) benign anastomotic stricture after oesophagojejunostomy; 2) dilated jejunal blind pouch on the ipsilateral side of the proximal anastomosis; 3) history of surgery for lower esophageal squamous cell carcinoma; and 4) history of total gastrectomy for cardiac cancer.

Condition analysis revealed that the patient's core problem was severe benign anastomotic stricture combined with jejunal blind pouch dilation, which formed a vicious cycle—stricture led to obstruction of food and intestinal fluid discharge, inducing jejunal blind pouch dilation; the reflux of retained substances in the blind pouch further aggravated anastomotic mucosal inflammation, leading to worsening stricture. Treatment required simultaneously addressing both lesions to fundamentally improve symptoms.

### 2.4 Data collection and follow-up methodology

This retrospective case report was prepared by reviewing inpatient and outpatient medical records from Shenzhen Second People's Hospital. Additional follow-up information was obtained via structured telephone interviews and outpatient clinic visits as documented in the medical record. Consequently, follow-up data originated from different sources depending on the specific time point. Symptom severity, including dysphagia and reflux, was assessed retrospectively from clinical documentation and patient recall during telephone interviews. Laboratory values were extracted from medical records where available; values not documented in the clinical record were excluded.

## 3 Treatment process

### 3.1 Formulation of treatment plan

#### 3.1.1 All equipment specifications consolidated in dedicated 'Equipment and Device Specifications' subsection

OTSC (Nanjing MicroPort, Model HCCD-0-195-M-C, 12 mm opening diameter, 6 mm leg length, traumatic type); ESD knife (Anrui AMH-EK-O-2.41 × 800); balloon dilation catheter (Boston Scientific, M00558430, 15 mm maximum diameter); covered metal stent (Korea Sewoon Medical, BE-2008, 20 mm diameter × 80 mm length, nitinol frame with silicone membrane, flared ends); hemoclips (Olympus HX-610-135L, 11 mm opening width); endoscope (Olympus GIF-H290Z).

#### 3.1.2 A treatment plan was formulated based on the patient's condition

(1) Prioritize treatment of the jejunal dilated blind pouch to avoid infection or stent blockage caused by reflux of retained substances in the blind pouch after stent placement. (2) Adopt OTSC combined with jejunal mucosal ESD to close the blind pouch. ESD is used to precisely resect the mucosal tissue at the edge of the blind pouch to reduce its volume, and then OTSC is used to achieve mechanical closure to ensure the closure effect. (3) After the blind pouch is closed, a covered metal stent is placed at the anastomosis to support the strictured segment and maintain lumen patency. (4) Nutritional support and anti-infection treatment are strengthened during the perioperative period to reduce the risk of complications. The sequential strategy (blind pouch first, then stricture) was designed to prevent reflux contamination of the stent from retained blind pouch contents.

### 3.2 Preoperative preparation

(1) Nutritional support: after admission, the patient was given oral enteral nutrition preparations combined with parenteral nutritional support to correct hypoalbuminaemia and increase albumin to above 35 g/L. (2) Fasting and water deprivation: 6 h before surgery. (3) Preparation of drugs and equipment: OTSC, disposable mucosal incision knife, balloon dilation catheter and guide wire, covered metal stent, electronic gastroscope and other necessary instruments. (4) Ethical communication: the treatment plan, risks, and expected effects were fully explained to the patient and his family, and an informed consent form was signed.

### 3.3 Treatment implementation

Treatment was performed under general anesthesia (intravenous combined anesthesia) by experienced endoscopists and anaesthesiologists (video-the entire process).

Video documentation: the Supplementary Video 1 documenting the entire endoscopic procedure is deposited in the Frontiers Media Video Repository (accession number: [to be assigned upon acceptance]). The video is segmented into five key procedural phases with precise timestamps: (1) 00:00–00:55—Endoscopic view showing anastomotic stricture approximately 30 cm from the incisors, with a stricture diameter of approximately 0.7 cm; a jejunal blind pouch is visible adjacent to the stricture and the endoscope barely passes through; (2) 00:56–03:27—Endoscopic ESD mucosal resection of the esophagojejunal blind pouch, including submucosal injection, circumferential mucosal incision, and centripetal tissue resection to the muscular layer; (3) 03:28–05:30—OTSC deployment and mechanical closure of the blind pouch orifice, demonstrating dual-clip sequential placement at 45° and 30° angles with tissue grasping depth confirmation; (4) 05:31–08:50—Balloon dilation of the anastomotic stricture via guidewire placement, followed by delivery and release of the covered metal stent across the stricture; (5) 08:51–09:57—Post-procedural endoscopic confirmation of stent patency, OTSC clip position, and complete blind pouch closure; fluoroscopic verification of stent expansion and position. Each segment includes synchronized endoscopic and fluoroscopic views with anatomical landmark annotations, ensuring full compliance with the journal's video-augmented case report requirements.

**Figure 2:**
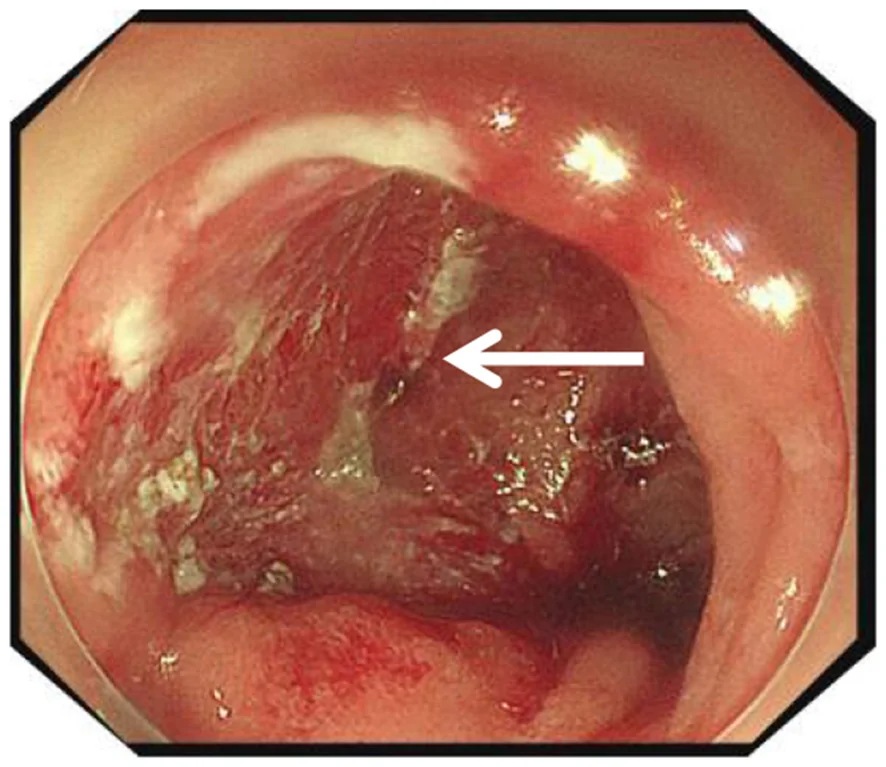
The white arrow indicates that a portion of the mucosa at the base of the dilated jejunal blind end was dissected.

**Figure 3:**
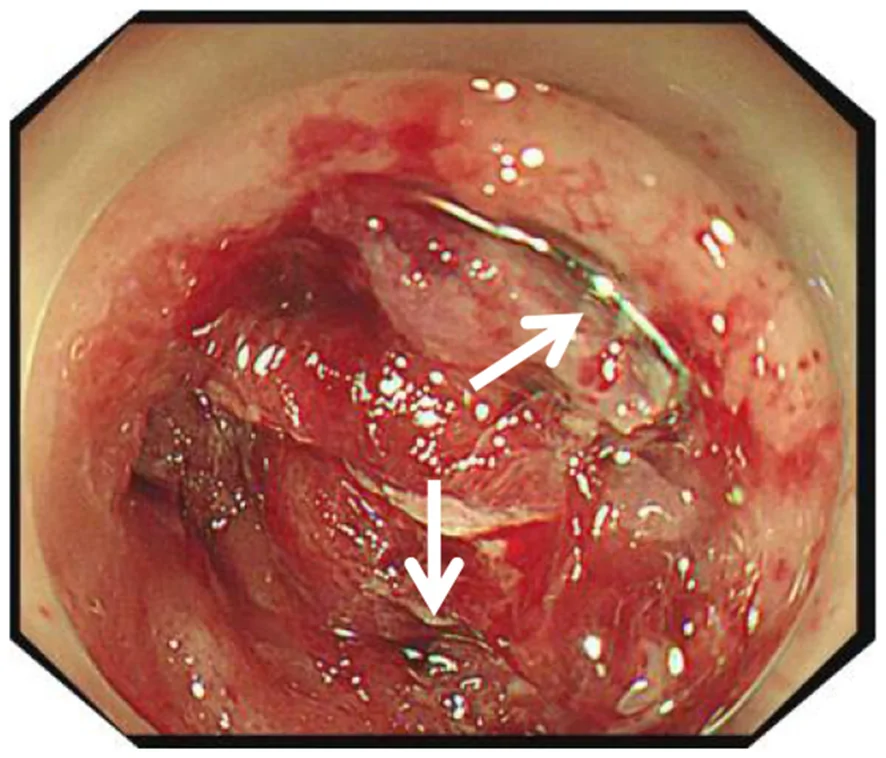
Two OTSCs were loaded into a special releaser and delivered to the orifice of the blind pouch through the working channel of the gastroscope. After adjusting the angle, the white arrow indicates that the OTSCs were released to close most of the orifice of the blind pouch.

Phase 1: closure of the jejunal dilated blind pouch. (1) ESD pretreatment (Figure 2): submucosal injection (0.9% normal saline 15 mL + indigo carmine 0.4 mL + 0.4% sodium hyaluronate 8 mL, total volume 23.2 mL) was administered at the orifice and inner wall of the blind pouch to fully separate the mucosa from the muscular layer, forming a “liquid cushion” with a height of 3–5 mm; a disposable mucosal incision knife was used to incise the mucosa along a circumferential marked line 2–3 mm from the pouch orifice, with electrocautery settings: ENDO CUT Q mode, effect 2, 80 W (ERBE VIO 300D), and the mucosal tissue on the inner side of the blind pouch was gradually resected in a centripetal direction to the bottom of the blind pouch, exposing the muscular layer and reducing the blind pouch volume by approximately 50% (resection area approximately 2.5 cm × 2.0 cm, based on pre-procedural CT volumetric assessment: original volume ~9.4 cm^3^, post-ESD residual volume ~4.7 cm^3^). Intraoperative blood loss was minimal (< 5 mL), with only small-vessel oozing observed. A small amount of oozing blood at the wound was closed with three hemoclips. Standardized hemostasis workflow: (1) Immediate bleeding site identification via irrigation and suction; (2) Sequential hemoclip deployment from proximal ESD wound margin, progressing distally in clockwise pattern; (3) Clip placement at 2-mm intervals along mucosal defect edge ensuring full-thickness approximation; (4) Final water immersion inspection confirming complete hemostasis. Specific clip sites: proximal, distal, and left lateral margins of blind pouch orifice. (2) OTSC closure (Figure 3): two OTSCs were sequentially loaded into a special releaser and delivered to the orifice of the blind pouch through the working channel of the gastroscope. The first OTSC was placed at the superior aspect of the blind pouch orifice at a 45° angle relative to the longitudinal axis of the jejunum, grasping approximately 1.5 cm of tissue depth (including mucosa and muscularis propria) to close the upper two-thirds of the orifice. The second OTSC was placed at the inferior aspect at a 30° angle, grasping 1.2 cm tissue depth to close the remaining lower one-third. The tissue grasping range was 8–10 mm per clip, ensuring full-thickness tissue approximation without excessive tension.

**Figure 4:**
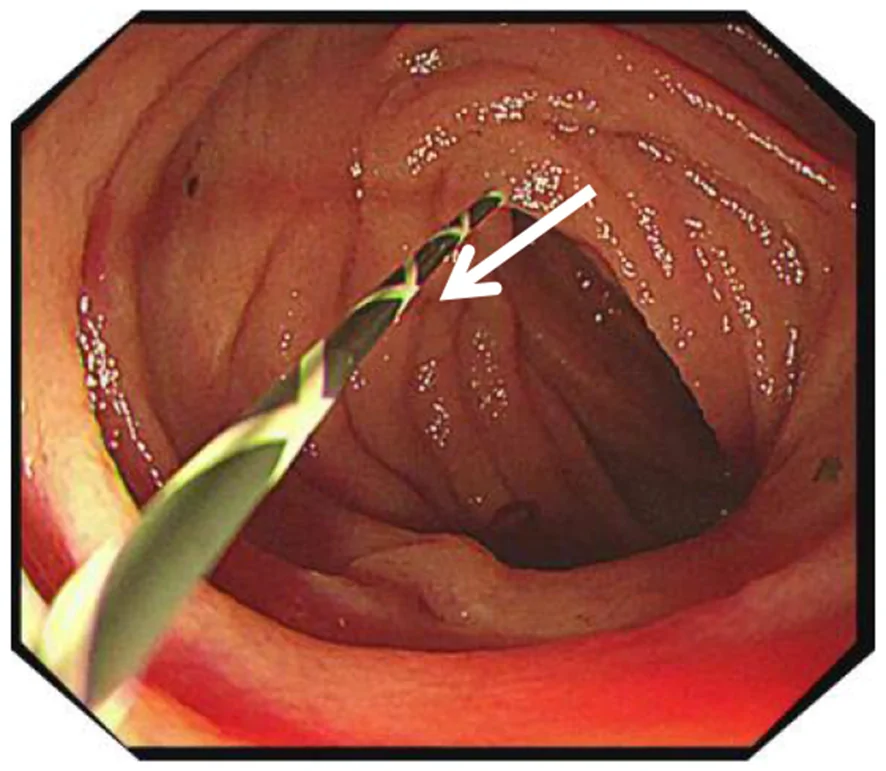
The white arrow indicates the guide wire, which was carefully passed through the stricture to the distal normal jejunal lumen to guide subsequent stent placement.

**Figure 5:**
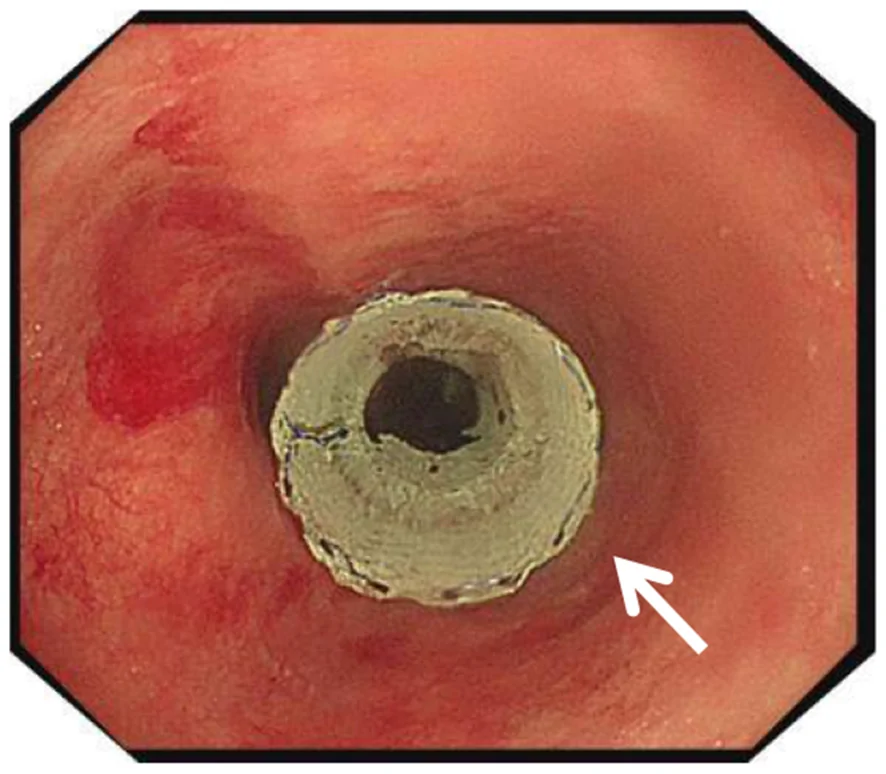
Endoscopically, the oral side of the stent is located in the esophagus, with a smooth surface and unobstructed lumen. The white arrow indicates the oral end of the stent.

**Figure 6:**
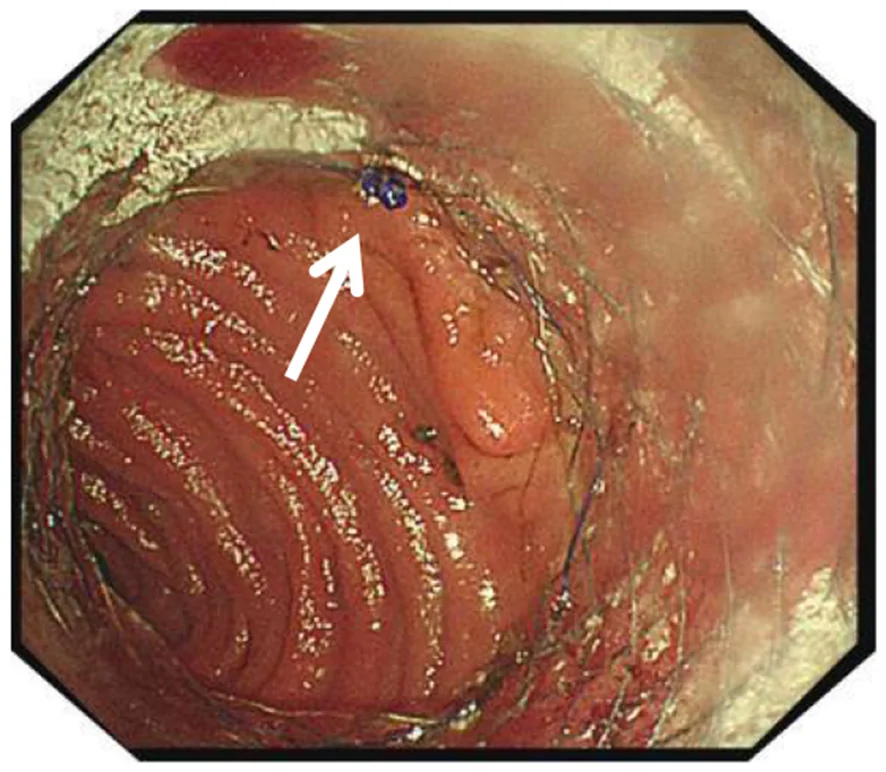
Endoscopically, the anal side of the stent is located in the jejunum, with a smooth surface and unobstructed lumen. The white arrow indicates the anal end of the stent.

**Figure 7:**
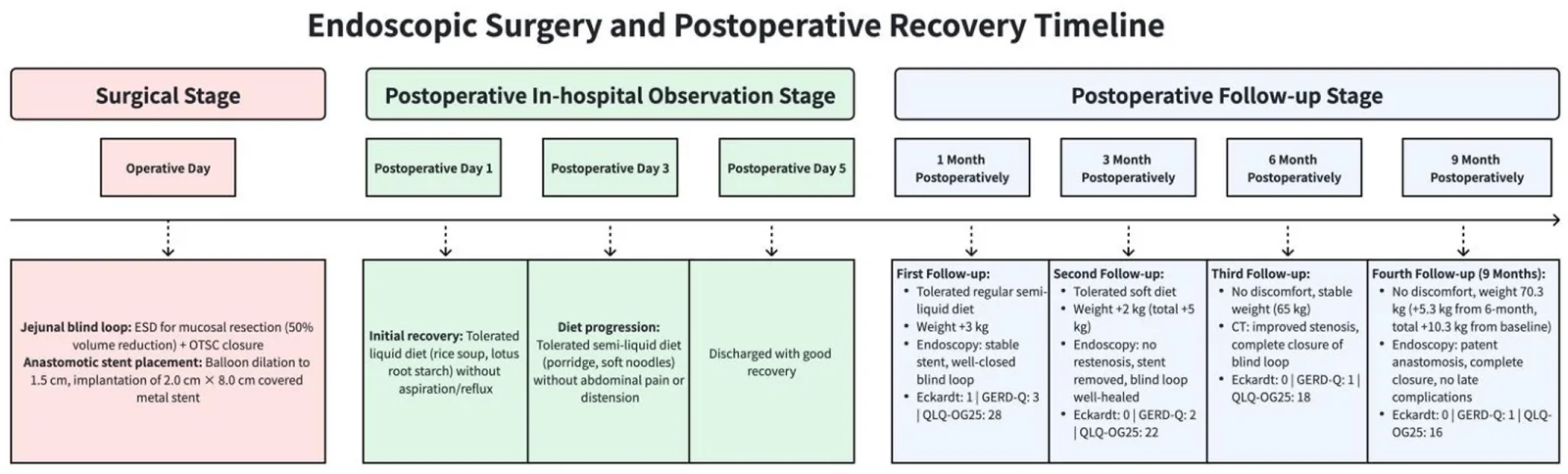
Endoscopic surgery and postoperative recovery timeline.

Phase 2: stent placement. (1) Balloon dilation: prior to stent placement, the stricture was dilated using a controlled radial expansion balloon. The balloon was positioned across the stricture under fluoroscopic guidance and inflated to 8 atm pressure for 3 min, followed by deflation and repeat inflation to 12 atm for 3 min (graded dilation protocol). The final stricture diameter was 1.5 cm. (2) Stent implantation (Figures 4–6): a fully covered self-expandable metal stent was selected based on the stricture length (2.4 cm) plus 2 cm proximal and 2 cm distal margins (total 6.4 cm covered by 80 mm stent). The guide wire was carefully passed through the stricture to the distal normal jejunal lumen to guide subsequent stent placement. The covered metal stent was delivered to the anastomotic stricture along the guide wire, ensuring that the proximal end of the stent was located in the dilated esophageal segment and that the distal end crossed the anastomosis into the jejunum by approximately 2 cm. After accurate positioning under combined endoscopic and fluoroscopic guidance, the stent was released, and 2 titanium clips were placed at the proximal flared end of the stent at the 2-o'clock and 10-o'clock positions to anchor the stent to the esophageal wall and prevent early displacement. (3) Postoperative confirmation: endoscopy revealed that the stent was in a good position, the lumen was patent, with no displacement or bleeding, and the endoscope was withdrawn (Figure 7-timeline).

## 4 Results

### 4.1 Immediate effects

After stent placement, endoscopy revealed that the lumen of the anastomotic stricture was patent; the OTSC at the jejunal blind pouch was fixed in position without displacement or leakage. On the first day after surgery, the patient consumed liquid foods such as rice soup and lotus root starch without choking or reflux; on the third day after surgery, he successfully consumed semiliquid foods such as porridge and mashed noodles without abdominal pain or distension; he was discharged from the hospital on the fifth day after surgery.

### 4.2 Follow-up

One-month follow-up after surgery: the patient could eat semiliquid food normally without obvious symptoms such as dysphagia, reflux, or abdominal pain. His weight increased by 3 kg compared with that at admission (BMI 19.9 kg/m^2^). Reexamination by gastroscopy revealed that the stent was stable in position without displacement or blockage, that the anastomotic mucosa was slightly congested without obvious hyperplasia, and that the lumen inside the stent was patent. The OTSC at the jejunal blind pouch was well fixed, with no ulcers or bleeding at the closed site. Routine blood and biochemical indicators returned to normal, and the serum albumin concentration increased to 38 g/L.

Three-month follow-up after surgery: the patient ate soft food normally without symptoms such as dysphagia, reflux, or abdominal pain. His weight increased by 2 kg compared with that 1 month after surgery (BMI 20.5 kg/m^2^). Reexamination by gastroscopy revealed that the stent was still in place, the anastomotic mucosa was smooth without signs of restenosis, and no obvious granulation tissue hyperplasia was observed at either end of the stent. The jejunal blind pouch was well closed with intact mucosa, and the OTSC was partially covered by mucosa without displacement. The covered metal stent was removed, and no obvious oozing blood was observed at the anastomosis after removal, indicating that the operation was successful. Histological biopsy after stent removal revealed chronic mucosal inflammation with focal squamous epithelial hyperplasia, mild submucosal fibrosis without excessive scar formation; immunohistochemistry (CK7, CK20, CDX2) confirmed benign squamous epithelial origin, supporting low restenosis risk.

Six-month follow-up after surgery: the patient had no obvious discomfort and could eat soft food, with a stable weight of 65 kg (BMI 20.5 kg/m^2^). Gastroscopy revealed that the anastomosis was patent, and the endoscope passed without resistance. The jejunal blind pouch was completely closed without dilation or effusion. Chest + abdominal CT indicated that the peritoneal adhesion was alleviated in comparison to earlier findings, and the stricture was relieved. The tumor marker levels were still within the normal range, with no signs of tumor recurrence.

Nine-month follow-up after surgery: the patient remained asymptomatic with no recurrent dysphagia, reflux, or abdominal pain. His weight had increased to 70.3 kg (BMI 22.2 kg/m^2^); this weight was self-reported during a structured telephone interview without independent verification, representing an additional 5.3 kg gain from the 6-month follow-up, total gain 10.3 kg from baseline. Gastroscopy confirmed patent anastomosis without restenosis, and the jejunal blind pouch remained completely closed without dilation or effusion. No late complications, including OTSC clip dislodgement, stent-related granulation tissue hyperplasia, or delayed perforation, were observed. Based on the telephone interview and retrospective review of clinical notes, the patient reported continued absence of dysphagia, minimal reflux, and good quality of life; no validated questionnaires were administered at this time point.

### 4.3 Complications

During the entire treatment and follow-up process, the patient did not experience serious complications. Only mild dull retrosternal pain occurred on the second day after stent placement, which was considered to have been caused by stent stimulation. Symptoms were relieved 3 days after symptomatic treatment; no infection, perforation, bleeding, stent blockage, or other complications occurred. No late complications were observed at the 9-month follow-up.

## 5 Discussion

The pathogenesis of benign anastomotic stricture after oesophagojejunostomy is complex and is related mainly to anastomotic technical defects (such as excessive tension at the anastomosis site and poor mucosal apposition), the local inflammatory response, excessive scar hyperplasia, and the patient's own scar constitution (16, 17). In this specific patient, multiple operative insults likely contributed to stricture formation: (1) the prior total gastrectomy with Roux-en-Y reconstruction altered the native gastrointestinal anatomy, resulting in abnormal jejunal peristaltic patterns and delayed emptying due to loss of gastric pacemaker function and vagal denervation; (2) the subsequent Ivor-Lewis esophagectomy with esophagojejunostomy created a second anastomosis in surgically manipulated tissue with compromised vascularity, particularly affecting the submucosal microcirculation essential for anastomotic healing; (3) the combination of two major reconstructions disrupted the enteric nervous system continuity and interstitial cell of Cajal networks, further impairing coordinated motility and predisposing to stasis-related complications. In this case, the patient developed progressive stricture 3 months after surgery, and pathology indicated chronic mucosal inflammation. Tumor recurrence was excluded; thus, it was considered to be a scar hyperplastic stricture caused by postoperative inflammation. The formation of the jejunal dilated blind pouch represents a pathophysiological cascade: anastomotic stricture → increased intraluminal pressure proximal to the obstruction → passive jejunal dilation → impaired peristalsis due to mural stretching and dysfunction of the enteric nervous system → stasis of intestinal contents → bacterial overgrowth and mucosal inflammation → further mucosal edema and stricture progression. This “stricture-blind pouch” vicious cycle is self-perpetuating and explains why single-lesion treatment strategies fail to achieve durable outcomes. This complex “stricture + blind pouch” situation is difficult to address with conventional treatment methods, making it a clinical challenge. Rationale for sequential treatment strategy (blind pouch first, then stricture): (1) Stent placement prior to blind pouch closure risks stent contamination and blockage from retained pouch contents (18); (2) Blind pouch closure first eliminates reflux source, optimizing stent patency (19); (3) Pathophysiologically, blind pouch digestive fluid and bacteria would reflux into esophagus after stent placement, promoting biofilm formation and aspiration pneumonia. Our sequential approach follows “treat the cause before relieving obstruction” principle.

Endoscopic balloon dilation is the initial treatment modality for simple anastomotic stricture. However, patients with severe stricture have a high recurrence rate after dilation. Studies have reported that the recurrence rate within 2 years is >50%, and repeated dilation may increase the risk of anastomotic tears and perforation (8, 9). Although bougie dilation is simple to perform, it has poor uniformity in dilating the strictured segment and is prone to causing mucosal damage (17). Owing to its strong support force and long patency maintenance time, metal stent placement has been increasingly used to treat severe stricture. In particular, covered stents can reduce stent blockage caused by mucosal hyperplasia, making them suitable for patients with benign stricture (20, 21). A covered metal stent was selected for this patient, and no stent blockage or obvious hyperplasia was observed 9 months after surgery, confirming its effectiveness. In this patient, two prior balloon dilations failed to maintain patency beyond 2–3 weeks, consistent with reported recurrence rates >50% within 2 years for severe strictures (8, 9). This treatment history underscores the limitation of single-modality therapy and justified the selection of combined stent placement.

The jejunal dilated blind pouch was the focus and was difficult to treat in this case. Traditional surgical treatment requires blind pouch resection + anastomotic reconstruction, which can cure the lesion but is associated with severe surgical trauma and slow recovery. In addition, the patient had a history of surgery for cardiac cancer and esophageal cancer, with a high risk of abdominal adhesion. Reoperation in patients with prior upper gastrointestinal surgery carries significant morbidity. Studies report anastomotic leak rates of 8–15%, wound infection rates of 10–20%, and prolonged ileus in 15–25% of cases following redo foregut surgery (12, 13). In this patient, the prior total gastrectomy and subsequent esophagectomy created extensive abdominal adhesions, estimated to increase operative time by 30–50% and conversion-to-open rates by 20–30% if surgical revision were attempted (12). Endoscopic treatment is the preferred minimally invasive approach, but its core lies in achieving effective closure of the blind pouch. As an “endoscopic suturing” tool, the OTSC pulls and closes tissues through mechanical force. Compared with traditional metal clips, it has a larger tissue grasping range and stronger closure tension and can be used for the repair of gastrointestinal defects with a diameter of ≤ 2 cm (14, 22). In this case, we first used ESD to resect the mucosal tissue on the inner side of the blind pouch. It reduces the volume of the blind pouch, shrinks the orifice of the blind pouch, and facilitates OTSC closure; conversely, mucosal resection exposes the muscular layer, increasing the firmness of tissue grasping and avoiding displacement after closure. Postoperative follow-up revealed complete closure of the blind pouch, confirming the feasibility of this combined method. To our knowledge, this is the first reported case of simultaneous endoscopic treatment of esophagojejunostomy stricture complicated by jejunal blind pouch using a triple-modality approach (ESD + OTSC + stent). Previously reported cases have focused on single-lesion management: OTSC alone for gastrointestinal perforations (14, 22), ESD alone for mucosal lesions (15), or stent alone for strictures (20, 21). Küllmer et al. (10) reported endoscopic jejunojejunostomy using a lumen-apposing metal stent for obstructed efferent loop, but this addressed mechanical obstruction rather than blind pouch closure. The novelty of our approach lies in: (1) the sequential treatment strategy (blind pouch first, then stricture) to prevent reflux contamination of the stent; (2) ESD-mediated volume reduction to enable OTSC closure of a large defect (>2 cm); and (3) the synergistic combination of three established techniques into a single-session procedure. Refined perioperative management reduces the risk of complications (23). Long-term OTSC retention: spontaneous clip shedding typically occurs within 6–12 months as tissue healing progresses (22). Potential risks include chronic mucosal irritation, focal ulceration, and delayed perforation. Extended follow-up at 12, 18, and 24 months is planned to monitor these complications.

Indications and contraindications for triple-modality regimen: indications—benign esophagojejunostomy stricture with jejunal blind pouch ≤ 4 cm, failed conventional dilation, no severe adhesions, normal coagulation. Contraindications—blind pouch >4 cm, severe adhesions precluding endoscopic access, coagulopathy (INR>1.5 or platelets < 501 × 0^9^/L), active infection, recurrent malignancy, intolerance to general anesthesia.

## 6 Conclusion

The treatment of severe anastomotic stricture complicated by a jejunal dilated blind pouch after oesophagojejunostomy is challenging, and single treatment modalities fail to simultaneously address both lesions. In this case, OTSC combined with jejunal mucosal ESD was used to precisely close the jejunal dilated blind pouch, and a covered metal stent was implanted at the anastomosis to relieve stricture, achieving the dual goals of “minimally invasive repair + patency maintenance.” The patient's digestive tract function significantly improved after surgery, with no serious complications or recurrence during follow-up, confirming the efficacy and safety of the treatment. This combined treatment regimen overcomes the limitations of traditional single treatment, providing an efficient and feasible new option for the clinical management of such complex postoperative complications, which is worthy of exploration and promotion in more clinical practice. However, its long-term value needs to be further confirmed by large-sample studies.

Nevertheless, this case has certain limitations: as a single-center case report, the sample size is small, and the long-term efficacy and safety require verification by more clinical studies; Second, data collection was retrospective: information was compiled from inpatient and outpatient medical records, with additional follow-up data at 9 months obtained via structured telephone interview. Consequently, follow-up data originated from different sources depending on the specific time point, and not all parameters were uniformly documented across visits. Third, the 9-month weight was self-reported during a telephone interview without independent verification, and no laboratory values (including serum albumin and prealbumin) were documented in the clinical record at 6- and 9-month follow-up; therefore, the nutritional trajectory beyond 1 month is incompletely documented. Fourth, the high cost of OTSC may limit its widespread application; and the applicability of this method for patients with severe intestinal adhesion or excessively large blind pouch diameters needs further exploration. Additional limitations include: (1) Preliminary evidence: the findings represent preliminary experience rather than established efficacy; (2) Technical complexity: the combined procedure requires advanced endoscopic skills including ESD expertise, OTSC deployment proficiency, and stent placement experience. The learning curve is estimated at 30–50 supervised cases for each component technique, limiting generalizability to non-specialized centers. The technical threshold and challenges for widespread promotion are significant. (3) Cost considerations: OTSC devices are expensive (approximately $1,000–1,500 per clip), which may restrict widespread adoption, particularly in resource-limited settings. (4) Long-term stent-related complications: while no granulation tissue hyperplasia or stent migration was observed at 9 months, the risk of late restenosis after stent removal (10–25% at 12 months in benign strictures (24, 25)) and potential for delayed OTSC clip dislodgement or perforation require extended follow-up. (5) Anatomical constraints: blind pouches >4 cm in maximum diameter or located at sharp angulations (>60° from the jejunal axis) may preclude effective OTSC closure due to insufficient tissue grasping or excessive tension on the closure site. The failure risks under these anatomical variations are not yet defined. (6) Cumulative mucosal injury: three endoscopic interventions (ESD, OTSC deployment, and stent placement) in a single session may cause cumulative thermal and mechanical mucosal damage. The long-term impact on anastomotic integrity, scar formation, and cancer surveillance requires careful evaluation in future studies. (7) The current 9-month follow-up demonstrates sustained efficacy, but longer observation is still required to fully exclude late recurrence. We have established a structured follow-up protocol at 12, 18, and 24 months post-procedure, incorporating serial Eckardt dysphagia scores, GERD-Q scores, EORTC QLQ-OG25 questionnaires, endoscopy, and CT imaging to comprehensively evaluate long-term outcomes, including potential late complications such as OTSC clip dislodgement, delayed perforation, and stent-related granulation tissue hyperplasia. In the future, multi-center, prospective studies should be conducted to expand the sample size, clarify the indications and contraindications of this combined treatment regimen, optimize the operation process, and explore more economical and efficient endoscopic devices to improve the accessibility and efficacy of treatment.

## Data Availability

The datasets presented in this study can be found in online repositories. The names of the repository/repositories and accession number(s) can be found in the article/Supplementary material.
